# IPM chemotherapy in cytokine refractory renal cell cancer

**DOI:** 10.1038/sj.bjc.6600934

**Published:** 2003-05-13

**Authors:** J Shamash, J P Steele, P Wilson, M Nystrom, W Ansell, R T D Oliver

**Affiliations:** 1 Department of Medical Oncology, St Bartholomew's Hospital, West Smithfield, London EC1A 7BE, UK

**Keywords:** cisplatin, irinotecan, mitomycin, renal cell carcinoma (RCC)

## Abstract

Renal cell carcinoma (RCC) is notoriously chemoresistant. Current management of metastatic disease usually includes immunological agents of which the most clearly evaluated is alpha interferon. Following the failure of such agents no clear second-line therapy exists. The use of a novel combination of cisplatin, irinotecan and mitomycin may offer some palliative benefit in this situation. Thirty-three patients with cytokine refractory RCC and documented progression and documented active progressive disease with performance status 0–3 were enrolled. Therapy consisted of cisplatin 40 mg m^−2^ on day 1 and day 15, irinotecan 100 mg m^−2^ on day 1 and day 15, and mitomycin 6 mg m^−2^ on day 1 of a 28-day cycle. The results showed that one patient (3%) had a partial response, eight (24%) had minor responses and nine (27%) had stable disease, overall 61% had symptomatic responses. Quality-of-life (QOL) assessment did not change significantly during therapy. Seventy-one percent of those who had primary refractory disease to cytokine therapy subsequently responded to IPM. The median progression-free interval was 4.8 months in this cohort on chemotherapy, compared to 3.9 months with their previous cytokine treatment. In conclusion, IPM produced symptomatic relief for a majority of patients with cytokine refractory RCC without any deterioration in QOL. Disease stabilisation on radiological assessment and symptomatic improvement were associated with prolonged survival. A degree of non-crossresistance to cytokine therapy was seen. IPM may be considered in patients with renal cancer following failure of cytokines.

Renal cell carcinoma (RCC) is a relatively common condition and when localised it is best treated by nephrectomy. Once the disease becomes metastatic, treatment options are limited. Current management has concentrated on the use of immunological agents, most commonly interferon alpha, which has shown a survival advantage when compared to medroxyprogesterone acetate in metastatic disease (Anonymous, 1999). The addition of interleukin-2 appears to improve the response rate but not the overall survival (Negrier *et al*, 1998). Nephrectomy in patients with metastatic disease appears to have an advantage over the use of interferon alone (Mickisch *et al*, 2001). To date, the most impressive results have been obtained with the combination of interferon alpha, interleukin-2 and fluorouracil, with response rate of 30–40% being reported (Allen *et al*, 2000; Atzpodien *et al*, 2001). Whether this treatment is a genuine advance over interferon alone in patients who are relatively fit is currently being examined by an MRC trial. The use of chemotherapy in RCC has been disappointing. It has been suggested that this may be because of the fact that renal cells produce large amounts of multidrug-resistance protein leading to the efflux of cytotoxics (Tobe *et al*, 1995; Reese *et al*, 2000). Chemotherapy in RCC has generally been based on either fluorouracil or its activated metabolite floxuridine (Reese *et al*, 2000). A recent report has demonstrated that the addition of gemcitabine may improve the response rate to about 20% (Rini and Durras, 2000).

The topoisomerase-1 inhibitor, irinotecan, has shown activity in renal xenografts (Miki *et al*, 1998). In other tumour types, synergy has been demonstrated with either cisplatin (Boku *et al*, 1999) or mitomycin C (Gil-Delgado *et al*, 2001). The combination of irinotecan, cisplatin and mitomycin C (IPM) is being evaluated at our institution in several tumour types and appears to show promising activity (Shamash *et al*, 2000). This was chosen for use in patients with biopsy-proven RCC following the failure of cytokine therapy. Only patients with progressive disease (PD) were treated; those with residual disease following cytokine therapy were observed until overt progression as it is clear that long periods of progression-free survival can be observed in the presence of metastatic disease and sometimes spontaneous regressions can be seen (Oliver *et al*, 1988).

In such an unresponsive tumour, objective response rate may not be a useful way of assessing activity: the use of time to progression and the symptomatic response rate may be more applicable and more important in judging the benefit of therapy. This paper reports the symptomatic and radiological responses as well as the time to progression following IPM chemotherapy.

## PATIENTS AND METHODS

Patients with histologically confirmed unresectable or metastatic RCC who were actively progressing despite cytokine-based therapy (interferon or interferon in combination with interleukin-2 and fluorouracil) with a performance status of 0–3 and a creatinine calculated clearance of >50 ml min^−1^ were eligible. Patients with cerebral metastases who were stabilised on steroids were also eligible.

### Statistics

The log-rank test was used to compare the progression-free interval and the overall survival between groups.

The Wilcoxon signed-rank test was used to look at the overall change in median scores of the quality-of-life (QOL) parameters.

### Study design

This was a phase II single institution study for which local research ethics committee approval was sought and given. The purpose of this trial was to assess the symptomatic and radiological response rate, as well as the progression-free survival in patients who had progressed following cytokine therapy. The progression-free survival was defined as the time from the initiation of therapy to the development of new symptoms attributable to PD with patients being followed up every month following the end of therapy. For pulmonary disease assessment, routine chest radiographs were ordered at every clinic visit; for other sites of disease routine scanning was not undertaken, but in the event of a new symptom developing an appropriate radiological investigation was performed, and if this confirmed PD the date of progression was taken as the date of this symptom and not the date of the scan.

The treatment consisted of outpatient chemotherapy on a 28-day cycle. On day 1, patients received oral granisetron 1 mg, dexamethasone 16 mg and frusemide 40 mg, followed by 1 l of 0.9% NaCl over 1 h, followed by bolus mitomycin C 6 mg m^−2^, irino-tecan 70–100 mg m^−2^ over 30 min (a lower dose being chosen if patients had performance status of 3 or had previous radiotherapy), and cisplatin 40 mg m^−2^ in 1 l 0.9% NaCl over 1 h. On day 15, the therapy was repeated without mitomycin C.

Dose reductions were made as follows. Treatment was given if the platelets were ⩾100 × 10^9^ l^−1^ and neutrophils ⩾1 × 10^9^ l^−1^, otherwise treatment was delayed by 1 week. Treatment could then be given if the neutrophils were >1 × 10^9^ l^−1^ and the platelets were ⩾75 × 10^9^ l^−1^ and rising. If the platelet count was ⩾30 and <75 × 10^9^ l^−1^ then the treatment was delayed a further week with mitomycin being given at 50% dose for all subsequent courses. If the platelet count was <30 × 10^9^ l^−1^, no further mitomycin was given. If neutropenic sepsis occurred requiring hospital admission, then a 20% dose reduction in all subsequent courses of cisplatin and irinotecan.

CT scans were repeated after two cycles (56 days of therapy) and if patients had no evidence of progression they proceeded to receive another two cycles of treatment followed by a further CT scan. A further two cycles of therapy without mitomycin could be given at the discretion of the treating physician. Patients were asked to fill in the EORTC QLQ C30 (+3) form prior to therapy and then at monthly intervals at the start of each new cycle of chemotherapy.

### Response assessment

Radiological responses were assessed as follows. Complete response defined as complete disappearance of radiological abnormalities, partial response (PR) defined as >50% decrease in the sums of the products of perpendicular diameters of all measurable lesions lasting at least 4 weeks. Progressive disease (PD) was defined as 25% increase in the sum of products of measurable lesions or the appearance of any new lesion or reappearance of any lesion that had disappeared. Minor response (MR) was used to define any tumour, which had radiologically reduced in size but had not met the criteria of a PR. Stable disease (SD) was defined as no evidence of radiological response or <25% increase in the sum of products of measured lesions in the absence of the appearance of any new lesions. Symptomatic response was defined as the disappearance of the symptom, for example, haemoptysis or pain previously attributed to progressive cancer for which no other therapy (e.g. analgesic) were considered responsible for.

### Patient characteristics

Thirty-three patients were treated between January 1999 and September 2001. Two patients were not evaluable for response. One patient stopped therapy after the first dose because of unacceptable toxicity, the other died of bronchopneumonia 14 days after starting therapy while neutropenic – both are included in the progression-free and symptomatic-response analyses (see Table 1

**Table 1:** Presentation characteristics

Number of patients	33	
Metastatic at diagnosis	14 out of 33	
Radical nephrectomy	21 out of 33	(inc. two with PA nodes)
Median interval radical surgery to commencing cytokines (in those 21 patients)	31.4 months	(1.6–92.8)
Male : female	29 : 4	
Median age	51	(27–70)
Hb		
<12	22	
>12	11	
WBC		
<10	23	
>10	10	
Platelets		
<450	22	
>450	11	
LDH		
<480	19	
>480	14	
PS		
0	5	
1	16	
2	8	
3	4	
No. metastatic sites		
1	12	
2	14	
3	7	
Median duration of disease	23.6 months	(3.1–162.4)
<2 years	16	
>2 years	17	
Median duration of metastases	15.6 months	(0.2–80.3)
<1 year	13	
>1 year	20	

).

## RESULTS

### Quality-of-life (QOL)

Twenty-four of 33 patients returned at least one QOL questionnaire. Fourteen completed a baseline and end of treatment form. The crude scores were converted into a scale of 0–100, where 100 is excellent QOL. The median, QOL score at the baseline was 62.5, this fell during therapy by a median of 8.5 (Wilcoxon's signed-rank test *P*=0.07). There was a slight increase in fatigue, nausea, vomiting and insomnia scores. All were statistically nonsignificant. No other changes in the side-effect scores occurred (see Table 2

**Table 2:** QLQ scores from start to end of treatment

		No. of patients			Change in
		Better	Same	Worse	score
QOL	Quality of life	3	3	8	−8.5
PF	Physical function	2	10	2	0
RF	Role function	6	6	0	0
EF	Emotional function	5	6	3	0
CF	Control function	1	7	6	0
SF	Social function	3	6	5	0
FA	Fatigue	1	5	8	+11.0
NV	Nausea and vomiting	2	5	7	+8.5
PA	Pain	3	8	3	0
DY	Dyspnoea	1	7	6	0
SL	Insomnia	2	5	7	+16.5
AP	Appetite	1	7	6	0
CO	Constipation	2	9	3	0
DI	Diarrhoea	1	8	5	0
FI	Financial difficulties	5	8		

).

### Response to therapy

The objective response to therapy was one out of 31 (3%). Eight patients (26%) had MR, nine(29%) had SD, 13 (42%) had PD. Twenty-eight had symptoms at the start of IPM, of whom 17 improved (61%). The overall progression-free interval (PFI) was 4.4 months compared to 3.9 months for the prior cytokine treatment (*P*=0.99). Those who had a symptomatic response had an improved PFI (6.2 *vs* 1.8 months, *P*=0.001). The median survival for all patients from the start of chemotherapy was 9.2 months. Thirteen (39%) were progression free at 6 months.

**Figure 1:**
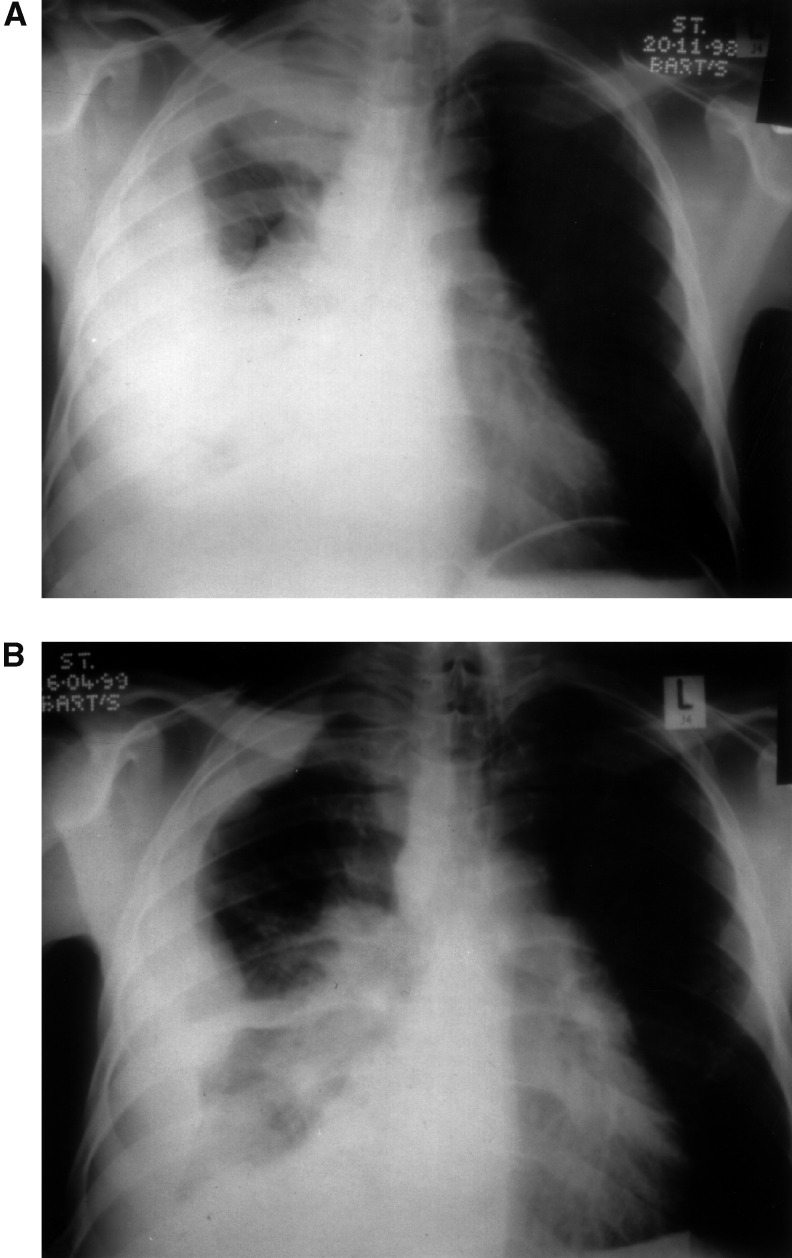
Chest radiograph prior and post-IPM therapy.

Those who had a symptomatic response had improved survival (10.8 *vs* 5.7 months, *P*=0.001), those who had radiologically stable disease or better also showed enhanced survival (14.3 *vs* 5.7 months for those with progressive disease, *P*=0.001) (see Figure 1

and Figure 2

**Figure 2:**
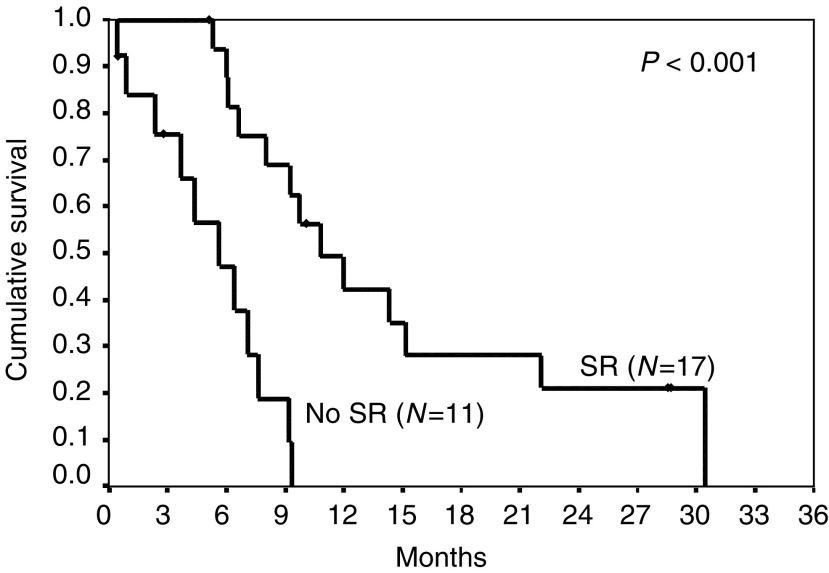
Overall survival by presence or absence of a symptomatic response.

).

### Comparison of IPM responses with prior response to cytokines

All patients had received prior cytokine therapy, 24 received interferon monotherapy and nine received triple therapy with interferon/interleukin-2/fluorouracil. There was no suggestion of any advantage for the triple therapy-treated patients (median PFI of 2.8 (triple therapy) *vs* 3.9 months). Fifteen out of 30 (50%) assessable patients (of the three nonassessable patients, two died prior to response assessment, one received cytokines in the adjuvant setting as part of a clinical trial) had at least SD to cytokines, as expected their PFI with cytokines was prolonged (10.2 *vs* 2.4 months, *P*=0.001). Similarly, those who had a symptomatic response (six out of 25, 24%) to cytokines had a trend to prolonged PFI with them (10.2 *vs* 3.7 months, *P*=0.09). Twenty-eight patients were evaluable for response for both cytokines and IPM. Fifteen had radiological progression during cytokines of whom nine (67%) subsequently had at least SD to IPM. Twenty-one of 28 had symptoms at the start of cytokines; of these, 15 did not have a symptomatic response to cytokines but 11 (73%) went on to have a symptomatic response to IPM, suggesting a lack of crossresistance between IPM and prior cytokine therapy (see Table 3

**Table 3:** Comparison between IPM response and response to previous cytokine therapy

Cytokines				
IFN monotherapy	24			
IFN combination therapy	9			
Median interval cytokines to commencing IPM	5.9 months		(1–62)	
				
	Cytokines	IPM		
Response				
Adjuvant	1		0	
PR	1		1	
MR	3		8	
SD	11		9	
PD	15		13	
NE	2		2	
PR or better	One out of 30 (3%)		One out of 31 (3%)	
SD or better	15 out of 30 (50%)		18 out of 31 (58%)	
Symptomatic				
Response	Six out of 25 (24%)		17 out of 28 (61%)	
				
*Median progression-free interval*				
Overall	3.9 months		4.4 months	
(a) SD or better	10.2 months		6.9 months	
(b) PD	2.4 months		1.9 months	
*P*-value (a *vs* b)	<0.001		<0.001	
(c) Symptomatic response	10.2 months		6.2 months	
(d) No symptomatic response	3.7 months		1.8 months	
*P*-value (c *vs* d)	0.09		<0.001	
				
*Symptomatic response to cytokines vs IPM*		Cytokine symptomatic response		
		No	Yes	Total
IPM symptomatic response	No	4	1	5
	Yes	11	5	16
	Total	15	6	21

).

### Toxicity

There was one treatment-related death, from bronchopneumonia while neutropenic. The most common grade 3–4 toxicity was malaise, which occurred in 17% of cycles. Neutropenia (19%) was frequently asymptomatic. There were 19 admissions for toxicity. Despite day case cisplatin with limited prehydration, there were no cases of severe nephrotoxicity (see Table 4

**Table 4:** Toxicity in IPM: by course, showing the percentage of courses complicated by WHO grade grade 3–4 toxicity and the number of patients who experienced this

	No. of courses	% Courses grade 3–4	No. of patients
Malaise	87	17	10
Dyspnoea	85	7	5
Infection	87	6	4
Diarrhoea	87	1	1
Anorexia	85	2	2
Nausea	87	1	1
Vomiting	86	0	0
Stomatitis	82	0	0
			
Neutropenia	89	19	12
Thrombocytopenia	90	1	1
Hb/WBC	90	10	5
Renal	80	0	0

Seventeen patients were admitted because of toxicity, of whom two were admitted on two occasions.

).

### Prognostic factors

**Figure 3:**
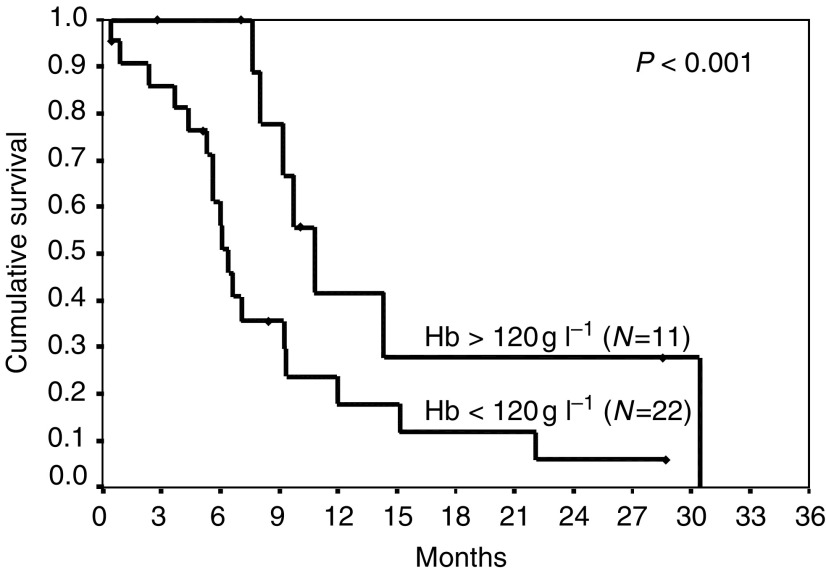
Overall survival by haemaglobin greater than or less than 120 g l^−1^.

The following pretreatment prognostic factors were studied: age, sex, performance status, white blood cell count, platelets, haemoglobin, lactate dehydrogenase, number of metastatic sites, presence of liver or central nervous metastases *vs* the presence of lung/nodal metastases only; with the exception of haemoglobin none had prognostic significance. A pretreatment haemoglobin of >120 g l^−1^ predicted for enhanced overall survival –see Figure 3

(10.8 *vs* 6.4 months, *P*=0.03).

## DISCUSSION

The study shows a moderate level of activity from IPM chemotherapy in patients with RCC who would normally be considered refractory to all current treatment. The number of patients experiencing symptomatic benefit was encouraging as was the fact that the progression-free survival was equal to that of the trial cytokine treatment in these patients (although the numbers were small) was also of note. The number progression free at 6 months was at least comparable to that seen with cytokines and the lack of crossresistance to cytokines is clearly interesting.

Patients with responses of SD or greater or those experiencing a symptomatic response, clearly had longer progression-free and overall survival. Interestingly, one patient who had a symptomatic response from a vertebral metastases leading to cord compression and paraplegia despite radiotherapy, improved sufficiently on chemotherapy to be able to regain the ability to walk and for his steroids to be reduced to a maintenance dose of 5 mg of prednisolone a day; however, as he developed a new lung lesion during this time, he had to be classed as having had PD.

The level of haemoglobin was the only prior investigation, which had prognostic value. This has also been seen with cytokines in RCC –indeed a raised haemoglobin appears to confer an even better prognosis (Janik *et al*, 1993). The reason for this is unclear and not related to erythropoietin levels (Ljungberg *et al*, 1992).

Responses seem independent of pre-existing performance status, duration of disease, number of metastatic sites. Quality-of-life analysis appeared to show that overall there was little change in QOL from therapy. Unfortunately, only two-thirds of patients completed at least two QOL forms, which clearly reduced the impact of this tool. In particular, it would have been useful to know what happened to QOL in the months following the end of chemotherapy. Those who had a symptomatic response and therefore much greater survival appeared to have similar QOL on treatment as those who did not respond at all. It can be concluded that a trial of two cycles of therapy in this patient group can be considered without a detrimental effect on QOL.

It has been recently shown that nephrectomy in patients with metastatic disease offered a survival advantage over cytokine therapy alone (Mickisch *et al*, 2001). A majority of patients in this group already had nephrectomies; whether palliative nephrectomy either post or prior to chemotherapy would improve things further remains to be determined.

To date the most impressive report of chemotherapy in renal cancer has used the combination of fluorouracil and gemcitabine. The partial response rate was 17% (Rini and Durras, 2000), not all patients had prior cytokine therapy, and it is not clear that all the patients were actively progressing at the time of treatment and this makes the interpretation of the impressive 28 week progression-free survival more difficult, especially as the overall median survival was 11.6 months (compared to 9.6 months in this study). The decision to observe patients with stable metastases rather than give immediate therapy contributed to the relatively long duration of metastatic disease prior to treatment in this cohort. This may be of importance in studies of renal cancer where long periods of time with stable metastatic disease can be encountered.

Allogeneic transplantation has been proposed as an attractive therapy for cytokine refractory RCC (Childs *et al*, 2000). The requirement for a matched sibling donor clearly restricts the potential of this approach as does the observation that it takes approximately 3 months to have any significant graft *vs* tumour effect to be evident. Patients with rapidly PD would not be suitable. This procedure may be considered in patients progressing despite cytokines, if they have stabilised during chemotherapy.

Alternative approaches have included the use of thalidomide; although its effect on this disease remains unclear, occasional dramatic responses have been seen (Stebbing *et al*, 2001). It has been suggested that this works as an antiangiogenic agent, although direct proof of this is lacking. In this cohort following IPM, seven patients went on to receive thalidomide; however, all but one progressed rapidly on it.

This study illustrates some of the problems in evaluating therapy in metastatic renal cancer. The objective response rate was low, but the improvement in symptoms and progression-free survival was encouraging, particularly as all patients had been progressing prior to therapy being started.

Future studies aiming to integrate cytokine therapy with chemotherapy, or fluorouracil or gemcitabine into an irinotecan-based regimen are clearly attractive as synergy has been demonstrated in other tumour types, for example, melanoma, where recently the advantages of biochemotherapy over standard cytotoxic therapy in terms of objective response and time to progression have been confirmed in a multicentre Phase III study (Eton *et al*, 2002). Such approaches may be particularly helpful in those predicted to have poor responses to cytokines alone, namely those with sarcomatoid tumours and those with a poorer performance status.

In conclusion, IPM chemotherapy offers modest but real symptomatic benefits in cytokine refractory renal cancer. Those with a normal level of haemoglobin prior to therapy appear to benefit the most, and those patients who have symptomatic responses or radiological stabilisation appear to have prolonged survival, the progression-free interval is comparable to the prior cytokine therapy and a degree of non-crossresistance has been demonstrated.
